# The neurological phenotype of developmental motor patterns during early childhood

**DOI:** 10.1002/brb3.1153

**Published:** 2018-11-28

**Authors:** Marieke J. Kuiper, Rick Brandsma, Roelineke J. Lunsing, Hendriekje Eggink, Hendrik J. ter Horst, Arend F. Bos, Deborah A. Sival

**Affiliations:** ^1^ Department of Neurology University Medical Center Groningen, University of Groningen Groningen The Netherlands; ^2^ Department of Neonatology, Beatrix Children’s Hospital University Medical Center Groningen, University of Groningen Groningen The Netherlands; ^3^ Department of Pediatrics, Beatrix Children’s Hospital University Medical Center Groningen Groningen The Netherlands

**Keywords:** children, development, motor behavior, movement disorders, phenotype

## Abstract

**Introduction:**

During early childhood, typical human motor behavior reveals a gradual transition from automatic motor patterns to acquired motor skills, by the continuous interplay between nature and nurture. During the wiring and shaping of the underlying motor networks, insight into the neurological phenotype of developmental motor patterns is incomplete. In healthy, typically developing children (0–3 years of age), we therefore aimed to investigate the neurological phenotype of developmental motor patterns.

**Methods:**

In 32 healthy, typically developing children (0–3 years), we video‐recorded spontaneous motor behavior, general movements (GMs), and standardized motor tasks. We classified the motor patterns by: (a) the traditional neurodevelopmental approach, by Gestalt perception and (b) the classical neurological approach, by the clinical phenotypic determination of movement disorder features. We associated outcomes by Cramer's V.

**Results:**

Developmental motor patterns revealed (a) choreatic‐like features (≤3 months; associated with fidgety GMs (*r* = 0.732) and startles (*r* = 0.687)), (b) myoclonic‐like features (≤3 months; associated with fidgety GMs (*r* = 0.878) and startles (*r* = 0.808)), (c) dystonic‐like features (0–3 years; associated with asymmetrical tonic neck reflex (*r* = 0.641) and voluntary movements (*r* = 0.517)), and (d) ataxic‐like features (>3 months; associated with voluntary movements (*r* = 0.928)).

**Conclusions:**

In healthy infants and toddlers (0–3 years), typical developmental motor patterns reveal choreatic‐, myoclonic‐, dystonic‐ and ataxic‐like features. The transient character of these neurological phenotypes is placed in perspective of the physiological shaping of the underlying motor centers. Neurological phenotypic insight into developmental motor patterns can contribute to adequate discrimination between ontogenetic and initiating pathological movement features and to adequate interpretation of therapeutic interactions.

## 1 INTRODUCTION

During the first three years of life, typically developing infants and toddlers show a gradual transition from innate motor patterns to acquired motor skills by the continuous interplay between nature and nurture (Teulier, Lee, & Ulrich, 2015). During the first year of life, key dynamic transitions induce the gradual replacement of innate neonatal motor patterns by goal‐directed movements (Einspieler & Prechtl, 2005). Until now, clinical insight into the neurological phenotype of these developmental motor patterns is still incomplete. We reasoned that neurological data on the phenotypic expression of the underlying developmental motor patterns would contribute to (a) insight into the functional developmental condition of the underlying developing motor centers and networks, (b) clinical neuro‐pediatric discrimination between physiological and pathological movement disorder features, (c) adequate phenotypic interpretation of therapeutic effects. In the present study, we therefore aimed to elucidate the neurological phenotype of developmental motor patterns by associating two different approaches: (a) the traditional neurodevelopmental approach, by the technique and theory of Gestalt Perception (Prechtl, 1990) and (b) the classical neurological approach, by the clinical phenotypic determination of movement disorder features.

The first traditional neurodevelopmental approach involves the assessment of the developmental motor patterns by Gestalt perception (Prechtl, 1990). This method describes the quality (i.e., variability in amplitude, speed, fluency, and symmetry) of spontaneous motor behavior, including general movements (GMs; (Prechtl, 1990)). GMs are complex, spontaneous movements, involving the whole body, characterized by variability in intensity, force, speed, and amplitude (Prechtl & Hopkins, 1986). During the early neonatal period, GMs are of writhing character (i.e., small‐to‐moderate amplitude and slow‐to‐moderate speed), transforming into fidgety quality (i.e., continuous small movements of moderate speed and variable acceleration of trunk, neck, and limbs in all directions) around six to nine weeks postterm (Einspieler & Prechtl, 2005; Prechtl & Hopkins, 1986; Prechtl, 1991). At about 20 weeks of age, fidgety GMs are gradually being displaced by intentional movements, involving grasping, rolling, sitting, and walking. During the acquisition of new motor patterns, the healthy motor system explores different strategies, resulting in variable motor output of optimal complexity (Dusing, Thacker, Stergiou, & Galloway, 2013). In this period, the nervous system is being shaped and organized by innate activation of neural circuitry and environmental interaction. These processes will result in the elimination of inefficient synaptic connections, preserving the most efficient neural networks (Edelman, 1993; Nishiyori, Bisconti, Meehan, & Ulrich, 2016). This organization concurs with a gradual change in the quality of motor behavior, changing from a clumsy pattern with co‐contractions, into fluent, precise, and well‐coordinated motor performances (Hempel, 1993a, 1993b; Jovanovic & Schwarzer, 2017; Largo, Fischer, & Rousson, 2003; Lin & Nardocci, 2016; Nishiyori et al., 2016).

The second classical neurological approach is based on the identification of movement disorder features by the examination of reflexes, postures, and movements. Historically speaking, this method is generally extrapolated from adult neurology. However, in early childhood it is important to realize that the neurological phenotype of immature, healthy motor networks could physiologically express movement disorder‐like features as part of normal neurological development. For instance, in healthy children older than four years of age, we have indicated that physiologically immature motor behavior can reveal features that resemble ataxia and dystonia (Brandsma et al., 2014; Kuiper et al., 2016). These physiological, developmental features are inversely related with age, implicating the highest expression by the most immature motor centers, and the gradual disappearance until adolescence. Analogous to movement quality features (as described by the neurodevelopmental approach), this implies that neurological movement disorder phenotypes express the physiological maturation and fine‐tuning of neural motor networks between the basal ganglia, cerebral cortex, and cerebellum (Edelman, 1993; Gogtay et al., 2004; Lenroot & Giedd, 2006; Nishiyori et al., 2016). In infants and toddlers (0–3 years of age), we reasoned that the occurrence of physiological developmental movement disorder features may clinically complicate the early quantitative distinction between ontogenetic and pathologic motor features and the neurological interpretation of treatment strategies.

In healthy, typically developing children (0–3 years of age), we aimed to investigate the neurological phenotype of developmental motor patterns. We hypothesized that developmental motor patterns in the neonate and toddler would consistently reveal movement disorder features (such as chorea, myoclonus, dystonia, and ataxia). If so, these developmental motor patterns could be neurologically attributed to the physiological shaping and maturation of the underlying motor centers.

## 2 METHODS

### 2.1 Participants

The medical ethical committee of the University Medical Center Groningen, the Netherlands, approved the present study. In the absence of pre‐existing data, the present study is explorative in character. Analogous to previous studies determining age‐related influences on quantitative ataxia and dystonia rating scale scores, we included four children per age subgroup.

After receiving signed informed consent by the parents, we included 32 healthy, typically developing children, consisting of four children (two male, two female) per age subgroup (i.e., 0, 3, 6, 9, 12, 18, 24, and 36 months of age). Inclusion criteria were as follows: healthy children, full term, uneventful delivery, normal development, and achievement of age‐adequate motor milestones (Supporting Information Appendix S1). Exclusion criteria were as follows: perinatal asphyxia, neurological or skeletal disorders, and medication with known side effects on motor behavior. We recruited the children by open advertisement. We collected physiognomic data on length, weight, and head circumference. Parents completed a questionnaire regarding their educational level, see Supporting Information Table S1.

### 2.2 Procedure

We videotaped pediatric motor behavior in a quiet and alert behavioral state (state 4). For the children's comfort, parents were present during the recordings. In children of 0 to 24 months of age, we videotaped 5 min of spontaneous motor behavior, including at least two GMs (0–3 months of age), spontaneous posturing and/or voluntary movements (6–24 months of age). In 3‐year‐old children, we videotaped 2 min of spontaneous motor behavior and standardized motor tasks (such as reaching, sitting, and walking), see Supporting Information Table S2.

### 2.3 Neurodevelopmental assessment of motor behavior

In children between 0 and 3 months of age, AFB, neonatologist and co‐founder of the General Movements Trust, scored and analyzed the GMs according to Prechtl's method of Gestalt perception (Einspieler, Prechtl, Ferrari, Cioni, & Bos, 1997). The average duration per assessment was three minutes.

### 2.4 Phenotypic assessment of physiologic immature motor patterns

Five investigators (three pediatric neurologists and two MD PhD students in pediatric movement disorders) independently assessed the motor patterns for the neurological phenotypic appearance. The average duration per phenotypic assessment was 10 min. For this task, the assessors applied the definitions of movement disorder features as the gold standard (see Supporting Information Appendix S2). For the assessment form, see Supporting Information Appendix S3.

In each child, we calculated the percentage of observers who phenotypically recognized the same movement disorder features (i.e., the % movement disorder recognition). If the same movement disorder feature was indicated by the majority of observers (≥3/5 observers), we considered the indicated movement disorder feature as “reproducible.” Subsequently, we analyzed the occurrence of reproducible movement disorder features per age subgroup (0, 3, 6, 9, 12, 18, 24, and 36 months of age, *n* = 4/age subgroup). When the majority of children per age subgroup (≥2/4) revealed the same reproducible movement disorder features, the indicated features were processed as “main” movement disorder features for that particular age subgroup. This implies that main movement disorder features are indicated by the majority of the observers in the majority of children per age subgroup.

We determined inter‐observer agreement for the obtained main movement disorder features (between five assessors). Furthermore, we associated the percentage of main movement disorder features with the age of the subgroups and also with the identified developmental motor patterns, involving GM characteristics using Gestalt Perception (by AFB, expert and co‐founder of the GM trust) and the identification of primitive reflexes (startles and asymmetric tonic neck reflex [ATNR]) and voluntary motor patterns (such as sitting, standing, walking, reaching, and voluntary grasping).

### 2.5 Statistical analysis

We performed statistical analyses using PASW Statistics 20 for Windows (SPSS Inc, Chicago IL, USA). We assessed normality of the distribution of the neurological phenotypic outcomes (i.e., percentage of recognition), both graphically and with the Shapiro‐Wilk test. We determined inter‐observer agreement between observers by Gwet's agreement coefficient (Gwet's AC_1_) and interpreted the outcomes by criteria of Landis and Koch: AC_1_ <0.20: slight; 0.21 to 0.40: fair; 0.41 to 0.60: moderate; 0.61 to 0.80: substantial; >0.81: almost perfect (Landis & Koch, 1977). We correlated the percentage of the main movement disorder features with age by Pearson's r or by Spearman's rho (when outcomes were not normally distributed). Finally, we correlated the developmental motor patterns with the percentage of the main movement disorder features with Cramer's V. *p*‐Values of <0.05 (two‐sided) were considered to indicate statistical significance.

## 3 RESULTS

### 3.1 Phenotypic assessment of the immature motor patterns

In healthy children between 0 and 3 years of age, neurological phenotypic assessment revealed: choreatic, myoclonic, dystonic, and ataxic features as main movement disorder characteristics (for illustration, see video S1‐S4). Features resembling tremor, tics, and hypotonia were only incidentally observed in the minority of the children per age subgroup. We therefore excluded these features from further analysis. The inter‐observer agreement (Gwet's AC_1_) regarding the phenotypic identification of main movement disorder features revealed statistically significant coefficients (*p* < 0.001) of 0.459 for choreatic features (“moderate”), 0.771 for myoclonic features (“substantial”), 0.755 for dystonic features (“substantial”), and 0.682 for ataxic features (“substantial”).

### 3.2 Association between main movement disorder features and age

**Figure 1:**
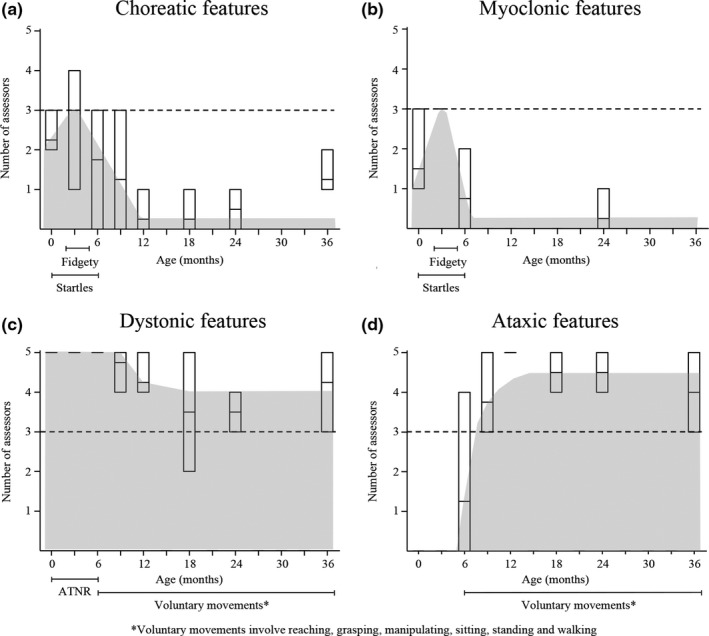
The recognition of movement disorder features per age subgroup. The recognition of movement disorder features per age subgroup. Boxes represent the minimum, mean, and maximum number of assessors who recognized the movement disorder feature per age group. Choreatic and myoclonic features coincide with startles and fidgety, dystonic features coincide with asymmetric tonic neck reflex and voluntary movements and ataxic features coincide with voluntary movements (>6 months of age)

In healthy children between 0 and 3 months of age, choreatic, myoclonic, and dystonic features were present in respectively 50%, 63%, and 100% of the children. In healthy children between 6 and 36 months of age, dystonic features persisted in 96% of the children, and ataxic features were indicated in 88% of the children, see Figure 1. The observed choreatic, myoclonic, dystonic, and ataxic features correlated significantly (*p < *0.01) with age (*r* = −0.526, *r *=* *−0.708*, r *=* *−0.632, and *r = *0.727, respectively).

### 3.3 Association between neurodevelopmental and movement disorder phenotypes

In healthy children between 0 and 3 months of age, fidgety GMs and startles correlated significantly with choreatic (*r = *0.732*, p = *0.002 and *r = *0.687*, p = *0.005*,* respectively) and myoclonic features (*r = *0.878*, p < *0.001 and *r = *0.808*, p < *0.001, respectively). Asymmetric tonic neck reflex correlated significantly with dystonic features (*r = *0.641*, p = *0.004).

**Figure 2:**
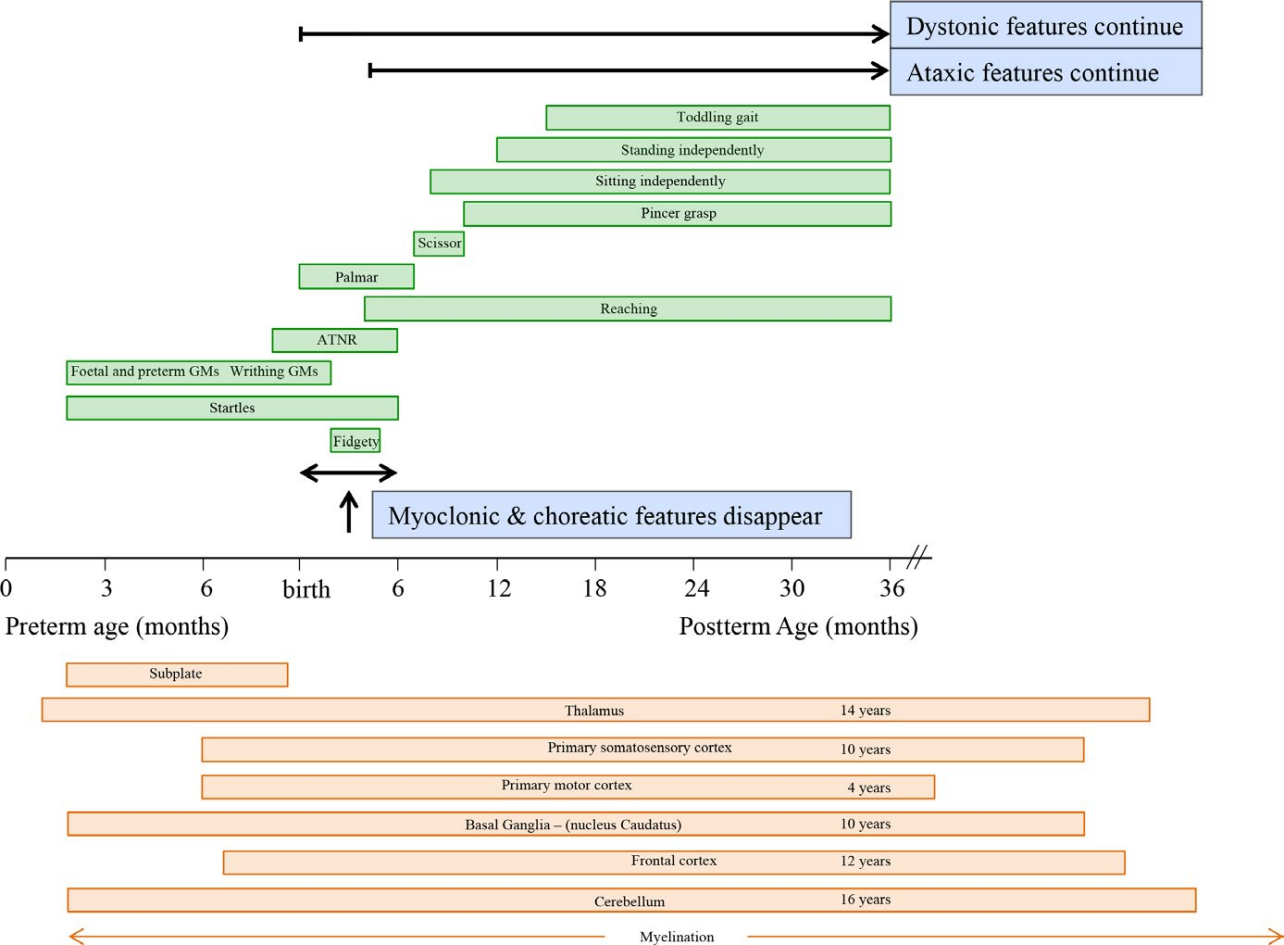
The timeline of developing motor patterns, movement disorder features and brain maturation. Green boxes indicate the normal age‐related presence of early neonatal movement patterns, primitive reflexes, and voluntary motor milestones. Blue boxes indicate the presence of physiological movement disorder features. Orange boxes indicate the maturation (determined by a peak in gray matter on MRI (Gogtay et al., 2004)) of developing motor centers. During development, normal ontogenetic motor behavior may reveal physiologic features resembling movement disorder characteristics

In healthy children between 6 and 36 months of age, the presence of voluntary coordinated movements correlated significantly with dystonic and ataxic features (*r = *0.517*, p = *0.036 and *r = *0.928*, p < *0.001*,* respectively). The correlation coefficients between voluntary motor patterns and neurological phenotypes are shown in Supporting Information Table S3. An overview of the concurrence between developmental motor patterns, the neurological phenotypic features, and physiological brain maturation is shown in Figure 2.

## 4 DISCUSSION

In the present study, we aimed to elucidate the neurological phenotype of developmental motor patterns. In infants (<3 months), developmental motor patterns (general movements and primitive reflexes) revealed hyperkinetic (choreatic, myoclonic, and dystonic) movement disorder features. Older children (6–36 months) were identified with persistent dystonic features and also with ataxic features during voluntary movements. In children of four years and older, these physiological developmental dystonic and ataxic features will gradually diminish and disappear during adolescence. The present discussion describes the transient occurrence of these motor features against the neurodevelopmental background of the underlying motor centers.

### 4.1 0 – 3 months of age

In healthy children between 0 and 3 months of age, hyperkinetic (choreatic, myoclonic, and dystonic) movement disorder features are physiologically present during the execution of developmental motor patterns. This is attributed to the development of the underlying motor centers and networks connecting the immature basal ganglia, cerebral cortex, and cerebellum. During the neonatal period, brain maturation involves many neurodevelopmental processes, including synaptic organization and myelination (Volpe, 2008). Synaptic organization involves synaptogenesis and subsequent synaptic pruning, peaking during the first 2 years of life (Ismail, Fatemi, & Johnston, 2017). This early period coincides with a “switch” in CNS receptors, due to the transition from excitatory to inhibitory GABA_A_ receptors and the functional activation of glutamatergic receptors (NMDA and AMPA)(Ben‐Ari, 2002; Ben‐Ari, Khazipov, Leinekugel, Caillard, & Gaiarsa, 1997; Ismail et al., 2017; Zhang & Poo, 2001). As this transition concurs with synaptic organization, these CNS receptors are considered to participate in the formation of the neural networks (Chugani, 1998; Ismail et al., 2017; Zhang & Poo, 2001). At three months of age, these neural networks reveal a significantly increased connectivity of the basal ganglia, cerebral cortex, and cerebellum (Chugani, 1998). This critical period concurs with the replacement of GMs and primitive reflexes by voluntary goal‐directed movements, social smiling, binocular vision, and stable state regulation (Feigelman, 2011; Volpe, 2008). Within this specific time frame, we also observed the disappearance of myoclonic and choreatic hyperkinetic movement disorder features. From the neurodevelopmental perspective, it is tempting to speculate that the disappearance of these hyperkinetic features from the neurological phenotype is related to enhanced inhibition by increased cortical activity (Sanger, 2003). Additionally, one could also speculate that increased functional activity of the basal ganglia (via the indirect and hyperdirect pathway) is related (Mink, 2003; Singer, Mink, Gilbert, & Jankovic, 2010). Altogether, our data indicate that neonatal myoclonic and choreatic movement disorder features are transiently present until the third month of age.

### 4.2 6 – 36 months of age

In children of six months and older, the process of synaptic organization continues to peak until the second year of life (Chugani, 1998; Ismail et al., 2017). During this period, the child achieves and subsequently refines voluntary functional motor performances, such as reaching, grasping, manipulation, sitting, standing, and walking (Fragaszy, Simpson, Cummins‐Sebree, & Brakke, 2016; Hempel, 1993a, 1993b; Yang, Mitton, Musselman, Patrick, & Tajino, 2015). In contrast with the disappearing choreatic and myoclonic features, dystonic features are persistent in the neurological phenotype. These data confirm our previous study data in older children of 4–16 years of age, revealing the existence of dystonic features. In this study group (4–16 years of age), dystonic features were inversely related with age (i.e., the strongest expression in the youngest children) and disappeared around adolescence (Kuiper et al., 2016). Although speculative, the early presence of dystonic features, the prolonged continuation and the gradual disappearance (before adulthood), could be attributed to the continuous development and maturation of the basal ganglia and the connecting networks. Due to the redundancy of neurons and synaptic connections in early childhood, inefficient activation of muscles may induce co‐contractions and dystonic overflow movements (Fog & Fog, 1963; Kuiper et al., 2016; Largo et al., 2007; Lin & Nardocci, 2016; Nishiyori et al., 2016). By the interaction between somatosensory and visual input and by selective elimination of inefficient synapses, basal ganglia neural networks will become more effective (Chugani, 1998; Edelman, 1993; Gogtay et al., 2004; Nishiyori et al., 2016), eventually resulting in the gradual disappearance of dystonic features from the neurological phenotype (Kuiper et al., 2016).

Analogous to the dystonic movement features at six months of age, we also observed that the neurological phenotype of voluntary movements reveals ataxic features. In a previous study, we have also shown that these physiologic ataxic features are persistent after 36 months, revealing an inverse relationship with age (i.e., the strongest expression in the youngest children) to disappear around adolescence (Brandsma et al., 2014). The execution and learning of coordinated movement patterns are generally regarded as a cerebellar function (Ghez & Thach, 2000). Cerebellar development starts by nine weeks gestational age, with ongoing neuronal proliferation and migration throughout the first year of life (Lavezzi, Ottaviani, Terni, & Matturri, 2006; White & Sillitoe, 2013). From the 24th week of gestation onwards, cerebellar circuits are being formed between the brainstem, thalamus, cerebral cortex, and the spinal cord (Wang & Zoghbi, 2001; White & Sillitoe, 2013). These cerebellar networks receive, process, and adapt information for balance and for decision‐making regarding speed, force, and direction of intended movements. Throughout childhood, selective synaptic elimination and subsequent myelination of the persistent connections will continuously shape the cerebellar network activity, resulting in a relatively protracted development and achievement of functional optimality (Lenroot & Giedd, 2006; Saksena et al., 2008; Tiemeier et al., 2010).

Altogether, in early childhood, the neurological phenotype of typical developmental motor patterns may reveal physiological movement disorder features as an expression of ongoing neurodevelopment (Chugani, 1998; Lenroot & Giedd, 2006; Saksena et al., 2008; Taki et al., 2013). In healthy children, it is important to realize that these physiological developmental movement disorder features should not be confused with the existence of a pathological movement disorder. On the contrary, the observation of these developmental movement disorder features during the execution of otherwise complex, fluent and variable developmental motor patterns should be regarded as an integrative part of normal neurodevelopment. We hope that neurological awareness of these physiologically occurring neurological phenotypes can contribute to: (a) insight into the functional expression of the underlying developing CNS, (b) adequate differentiation between normal ontogenetic and initiating pathologic motor behavior, and (c) phenotypic interpretation of treatment interventions.

We recognize some limitations to this study. First, the included number of children is relatively small. However, the reported movement disorder features were consistent and statistically significant, despite the small numbers. Second, we are aware that we only processed the outcome parameters of the “main” movement disorder features, as we strived to illuminate the consistent expression of the developing motor networks. This implies that other, less dominant, movement disorder features could still incidentally be observed as a physiological expression of the developing motor centers during early childhood.

In conclusion, in typically developing infants and toddlers, transient movement disorder phenotypes are attributed to physiological neurodevelopment. Neurological phenotypic insight into developmental motor patterns may hopefully contribute to adequate discrimination between ontogenetic and initiating pathological movement features and to adequate interpretation of therapeutic interventions.

## CONFLICT OF INTEREST

None declared.

## References

[brb31153-bib-0001] Ben‐Ari, Y. (2002). Excitatory actions of gaba during development: The nature of the nurture. Nature Reviews Neuroscience, 3(9), 728–739. 10.1038/nrn920 12209121

[brb31153-bib-0002] Ben‐Ari, Y. , Khazipov, R. , Leinekugel, X. , Caillard, O. , & Gaiarsa, J.‐L. (1997). GABAA, NMDA and AMPA receptors: A developmentally regulated ménage à trois’. Trends in Neurosciences, 20(11), 523–529. 10.1016/S0166-2236(97)01147-8 9364667

[brb31153-bib-0003] Brandsma, R. , Spits, A. H. , Kuiper, M. J. , Lunsing, R. J. , Burger, H. , Kremer, H. P. , … a., (2014). Ataxia rating scales are age‐dependent in healthy children. Developmental Medicine and Child Neurology, 56(6), 556–563. 10.1111/dmcn.12369 24392880

[brb31153-bib-0004] Chugani, H. T. (1998). A critical period of brain development: Studies of cerebral glucose utilization with PET. Preventive Medicine, 27(2), 184–188. 10.1006/pmed.1998.0274 9578992

[brb31153-bib-0005] Dusing, S. C. , Thacker, L. R. , Stergiou, N. , & Galloway, J. C. (2013). Early complexity supports development of motor behaviors in the first months of life. Developmental Psychobiology, 55(4), 404–414. 10.1002/dev.21045 22573386 PMC3496827

[brb31153-bib-0006] Edelman, G. (1993). Neural Darwinism: Selection and reentrant signaling in higher brain function. Neuron, 10, 115–125. 10.1016/0896-6273(93)90304-A 8094962

[brb31153-bib-0007] Einspieler, C. , & Prechtl, H. F. R. (2005). Prechtl’s assessment of general movements: A diagnostic tool for the functional assessment of the young nervous system. Mental Retardation and Developmental Disabilities Research Reviews, 11(1), 61–67. 10.1002/mrdd.20051 15856440

[brb31153-bib-0008] Einspieler, C. , Prechtl, H. F. R. , Ferrari, F. , Cioni, G. , & Bos, A. F. (1997). The qualitative assessment of general movements in preterm, term and young infants — review of the methodology. Early Human Development, 50(1), 47–60. 10.1016/S0378-3782(97)00092-3 9467693

[brb31153-bib-0009] Feigelman, S. (2011). The first year In Kliegman R., Behrman R., Jenson H., & Stanton B. (Eds.), Nelson textbook of pediatrics, 19th ed. (pp. 29–30). Philadelphia, PA: Elsevier Saunders.

[brb31153-bib-0010] Fog, E. , & Fog, M. (1963). Cerebral Inhibition Examined by Associated Movements In Bax M. & Mac Keith R. (Eds.), Minimal cerebral dysfunction, clinics in developmental medicine (pp. 52–57). London, UK: Heinemann Medical.

[brb31153-bib-0011] Fragaszy, D. , Simpson, K. , Cummins‐Sebree, S. , & Brakke, K. (2016). Ontogeny of tool use: How do toddlers use hammers? Developmental Psychobiology, 58(6), 759–772. 10.1002/dev.21416 27120556

[brb31153-bib-0012] Ghez, C. , & Thach, W. T. (2000). The Cerebellum BT ‐ Principles of Neural Science. *Principles of Neural Science*.

[brb31153-bib-0013] Gogtay, N. , Giedd, J. N. , Lusk, L. , Hayashi, K. M. , Greenstein, D. , Vaituzis, A. C. , … Thompson, P. M. (2004). Dynamic mapping of human cortical development during childhood through early adulthood. Proceedings of the National Academy of Sciences of the United States of America, 101(21), 8174–8179. 10.1073/pnas.0402680101 15148381 PMC419576

[brb31153-bib-0014] Hempel, M. S. (1993a). Neurological development during toddling age in normal children and children at risk of developmental disorders. Early Human Development, 34(1–2), 47–57. 10.1016/0378-3782(93)90040-2 8275882

[brb31153-bib-0015] Hempel, M. S. (1993b). The neurological examination for toddler‐age. (pp. 35–147), PhD‐thesis. Groningen, The Netherlands: University of Groningen..

[brb31153-bib-0016] Ismail, F. Y. , Fatemi, A. , & Johnston, M. V. (2017). Cerebral plasticity: Windows of opportunity in the developing brain. European Journal of Paediatric Neurology, 21(1), 23–48. 10.1016/j.ejpn.2016.07.007 27567276

[brb31153-bib-0017] Jovanovic, B. , & Schwarzer, G. (2017). The influence of grasping habits and object orientation on motor planning in children and adults. Developmental Psychobiology, 59(8), 949–957. 10.1002/dev.21573 29071707

[brb31153-bib-0018] Kuiper, M. J. , Vrijenhoek, L. , Brandsma, R. , Lunsing, R. J. , Burger, H. , Eggink, H. , … Sival, D. A. (2016). The Burke‐Fahn‐Marsden dystonia rating scale is age‐dependent in healthy children. Movement Disorders Clinical Practice, 3(6), 580–586. 10.1002/mdc3.12339 PMC6353340 30838251

[brb31153-bib-0019] Landis, J. R. , & Koch, G. G. (1977). The measurement of observer agreement for categorical data. Biometrics, 33(1), 159–174. 10.2307/2529310 843571

[brb31153-bib-0020] Largo, R. H. , Caflisch, J. A. , Hug, F. , Muggli, K. , Molnar, A. A. , & Molinari, L. (2007). Neuromotor development from 5 to 18 years. Part 2: Associated movements. Developmental Medicine & Child Neurology, 43(7), 444–453. 10.1111/j.1469-8749.2001.tb00740.x 11463174

[brb31153-bib-0021] Largo, R. , Fischer, J. , & Rousson, V. (2003). Neuromotor development from kindergarten age to adolescence: Developmental course and variability, (3200). (pp. 193–200). 10.4414/smw.2003.09883 12811675

[brb31153-bib-0022] Lavezzi, A. M. , Ottaviani, G. , Terni, L. , & Matturri, L. (2006). Histological and biological developmental characterization of the human cerebellar cortex. International Journal of Developmental Neuroscience, 24(6), 365–371. 10.1016/j.ijdevneu.2006.06.002 16893622

[brb31153-bib-0023] Lenroot, R. K. , & Giedd, J. N. (2006). Brain development in children and adolescents: Insights from anatomical magnetic resonance imaging. Neuroscience and Biobehavioral Reviews, 30(6), 718–729. 10.1016/j.neubiorev.2006.06.001 16887188

[brb31153-bib-0025] Lin, J.‐P. , & Nardocci, N. (2016). Recognizing the common origins of dystonia and the development of human movement: A manifesto of unmet needs in isolated childhood dystonias. Frontiers in Neurology, 7, 226 10.3389/fneur.2016.00226 28066314 PMC5165260

[brb31153-bib-0026] Mink, J. W. (2003). The basal ganglia and involuntary movements. Archives of Neurology, 60(10), 1365 10.1001/archneur.60.10.1365 14568805

[brb31153-bib-0027] Nishiyori, R. , Bisconti, S. , Meehan, S. K. , & Ulrich, B. D. (2016). Developmental changes in motor cortex activity as infants develop functional motor skills. Developmental Psychobiology, 58(6), 773–783. 10.1002/dev.21418 27096281

[brb31153-bib-0028] Prechtl, H. F. R. (1990). Qualitative changes of spontaneous movements in fetus and preterm infant are a marker of neurological dysfunction. Early Human Development, 23(3), 151–158. 10.1016/0378-3782(90)90011-7 2253578

[brb31153-bib-0029] Prechtl, H. F. (Ed.) (1991). Continuity of neural functions from prenatal to postnatal life. (pp. 179–197). Oxford, UK: Cambridge University Press.

[brb31153-bib-0030] Prechtl, H. F. , & Hopkins, B. (1986). Developmental transformations of spontaneous movements in early infancy. Early Human Development, 14(3–4), 233–238. 10.1016/0378-3782(86)90184-2 3803269

[brb31153-bib-0031] Saksena, S. , Husain, N. , Malik, G. K. , Trivedi, R. , Sarma, M. , Rathore, R. S. , … Gupta, R. K. (2008). Comparative evaluation of the cerebral and cerebellar white matter development in pediatric age group using quantitative diffusion tensor imaging. The Cerebellum, 7(3), 392–400. 10.1007/s12311-008-0041-0 18581196

[brb31153-bib-0032] Sanger, T. D. (2003). Pathophysiology of pediatric movement disorders. Journal of Child Neurology, 18(Suppl 1), S9–S24. 10.1177/0883073803018001S0401 13677568

[brb31153-bib-0033] Singer, H. S. , Mink, J. W. , Gilbert, D. L. , & Jankovic, J. (2010). In Brigido A., & Ball T. (Eds.), Movement disorders in childhood (pp. 2–7). Philadelphia, PA: Saunders Elsevier.

[brb31153-bib-0034] Taki, Y. , Hashizume, H. , Thyreau, B. , Sassa, Y. , Takeuchi, H. , Wu, K. , … Kawashima, R. (2013). Linear and curvilinear correlations of brain gray matter volume and density with age using voxel‐based morphometry with the Akaike information criterion in 291 healthy children. Human Brain Mapping, 34(8), 1857–1871. 10.1002/hbm.22033 22505237 PMC6870089

[brb31153-bib-0035] Teulier, C. , Lee, D. K. , & Ulrich, B. D. (2015). Early gait development in human infants: Plasticity and clinical applications. Developmental Psychobiology, 57(4), 447–458. 10.1002/dev.21291 25782975

[brb31153-bib-0036] Tiemeier, H. , Lenroot, R. K. , Greenstein, D. K. , Tran, L. , Pierson, R. , & Giedd, J. N. (2010). Cerebellum development during childhood and adolescence: A longitudinal morphometric MRI study. NeuroImage, 49(1), 63–70. 10.1016/j.neuroimage.2009.08.016 19683586 PMC2775156

[brb31153-bib-0037] Volpe, J. (2008). Neurology of the newborn (fifth). Philadephia, PA: Saunders Elsevier.

[brb31153-bib-0038] Wang, V. Y. , & Zoghbi, H. Y. (2001). Genetic regulation of cerebellar development. Nature Reviews Neuroscience, 2(7), 484–491. 10.1038/35081558 11433373

[brb31153-bib-0039] White, J. J. , & Sillitoe, R. V. (2013). Development of the cerebellum: From gene expression patterns to circuit maps. Wiley Interdisciplinary Reviews: Developmental Biology, 2(1), 149–164. 10.1002/wdev.65 23799634

[brb31153-bib-0040] Yang, J. F. , Mitton, M. , Musselman, K. E. , Patrick, S. K. , & Tajino, J. (2015). Characteristics of the developing human locomotor system: Similarities to other mammals. Developmental Psychobiology, 57(4), 397–408. 10.1002/dev.21289 25754858

[brb31153-bib-0041] Zhang, L. I. , & Poo, M. (2001). Electrical activity and development of neural circuits. Nature Neuroscience, 4(Supp), 1207–1214. 10.1038/nn753 11687831

