# Supplementary material for: The neurological phenotype of developmental motor patterns during early childhood
Source: Brain Behav. 2018 Nov 28;9(1):e01153. doi: 10.1002/brb3.1153 (PMC6346655; doi:10.1002/brb3.1153)
Supplement: Supplementary file 1 [file BRB3-9-e01153-s001.docx]

**Supplementary Table 1. Population characteristics**

|  | Girls (n = 16) | Boys (n = 16) | Total (n = 32) | Dutch pop. (%) |
| --- | --- | --- | --- | --- |
| Gestational age (weeks)  range  mean  Age at video recording (months)  range  mean (SD) | 37+0 – 41+0  38+5 (1+2)  0 – 36  14 (12) | 38+0 – 41+3  39+5 (1+0)  0 – 36  14 (12) | 37+0 – 41+3  39+2 (1+2)  0 – 36  14 (11) |  |
| Highest education achievement mother  higher education  vocational education  secondary school  missing value | 8 (50%)  4 (25%)  0 (0.0%)  4 (25%) | 9 (56.3%)  5 (31.3%)  0 (0.0%)  2 (12.5%) | 17 (53.1%)  9 (28.1%)  0 (0.0%)  6 (18.8%) | 25.9%  56.9%  16.9%  0.3% |
| Highest education achievement father  higher education  vocational education  secondary school  missing value | 7 (43.8%)  5 (31.3%)  0 (0.0%)  4 (25%) | 8 (50%)  6 (37.5%)  0 (0.0%)  2 (12.5%) | 15 (46.9%)  11 (34.4%)  0 (0.0%)  6 (18.8%) | 29.6%  54.8%  14.7%  0.9% |

Legends. pop. = population; Dutch population numbers were determined from Central Statistical Office of the Netherlands (CBS 2007); Parents of the included children had achieved academic grades more often (47 – 53%) compared to the average Dutch population (26 – 30%).

**Supplementary Table 2. Video protocol 3-year old children**

| General view | Walking | F – general view |
| --- | --- | --- |
| Sitting | Sitting at rest | F – general view &  P – general view |
|  | Eyes tracking movements | F – close-up |
|  | Eyes blinking (10x) | F – close-up |
|  | Opening and closing mouth | F – close-up |
|  | Tongue protrusion | F – close-up |
|  | Speech (counting 1-10 and normal conversation) | F – close-up |
|  | Head movements (rotation, lateroflexion and flexion/extension) | F – close-up |
|  | Elevate arms sidewards (5x) | F – general view |
|  | Finger to nose, right and left (5x) | F – general view |
|  | Drawing | F – general view |
| Lying position | Lying in rest | F – general view |
|  | Rolling | F – general view |
| Standing position | Stand upright | F – general view |

Legends. Duration of recording for each task is 30 seconds. F = frontal view; P = profile view.

**Supplementary** **Table 3. Association between voluntary movements and movement disorder features**

|  | Dystonic features | | Ataxic features | |
| --- | --- | --- | --- | --- |
|  | Correlation coefficient | Observed features | Correlation coefficient | Observed features |
| Sitting independently | 0.641* | Posturing of feet, arms, tongue | 0.943** | Trunk oscillations |
| Standing independently | 0.516* | Posturing of feet, arms, tongue | 0.649* | Broad base, trunk oscillations |
| Walking (toddlers gait) | 0.630* | Posturing of feet, arms, tongue | 0.570* | Broad base, variable steps |
| Reaching | 0.478 (ns)^#^ | Posturing of feet, arms, tongue | 0.856** | Dysmetria |
| Voluntary grasping | 0.478 (ns)^#^ | Posturing of feet, arms, tongue | 0.856** | Dysmetria |

Legends. The association (Cramer’s V correlation coefficients and the observed features) between various voluntary movements and dystonic and ataxic features in healthy children; Ataxic features were observed in the involved body region performing the voluntary movement, whereas dystonic features were observed in the whole body during all voluntary tasks (e.g. overflow, co-contraction); ^#^ Although the association between reaching and grasping and dystonic features was not significant, we did observe dystonic features of feet, arms and tongue. As the presence of the voluntary reaching and grasping (>4 months) concurs with a decrease in % of recognition (figure 1), this observation led to no statistic outcome. * *p < 0.05; ** p < 0.001*
