# Supplementary material for: The neurological phenotype of developmental motor patterns during early childhood
Source: Brain Behav. 2018 Nov 28;9(1):e01153. doi: 10.1002/brb3.1153 (PMC6346655; doi:10.1002/brb3.1153)
Supplement: Supplementary file 6 [file BRB3-9-e01153-s006.docx]

**Appendix S1. Description and age at presence of developmental motor patterns**

| **Developmental motor pattern** | **Description** | **Age** |
| --- | --- | --- |
| **Neonatal movements** |  |  |
| Writhing general movements | Gross movements involving the whole body in a variable and complex sequence, with small-to-moderate amplitude and slow-to-moderate speed.(Einspieler and Prechtl 2005; Prechtl et al. 1986) | 0 – 9 weeks |
| Fidgety general movements | Restless but smoothly rounded movements of the whole body with small amplitude, moderate speed and variable acceleration of neck, trunk and limbs in all directions.(Einspieler and Prechtl 2005; Prechtl et al. 1986) | 6 – 20 weeks |
| **Primitive reflexes** |  |  |
| Startle | A quick generalized movement, initiated in the limbs and spreading to neck and trunk, as a response to tactile, auditory and visual stimuli.(Volpe 2008; de Vries, Visser, and Prechtl 1982) | 0 – 6 months |
| Asymmetric tonic neck reflex (ATNR) | Extension of the upper extremity on the side to which the face is rotated and flexion of the upper extremity on the side of the occiput. Elicited by rotation of the head.(Volpe 2008) | 0 – 6 months |
| **Voluntary movements** |  |  |
| **Gross motor skills** |  |  |
| Rolling | Rolling over from supine into prone position, using rotation of the body on the pelvis.(Touwen 1976) | > 4.5 months |
| Wriggling and pivoting | Spatial displacement without use of arms and/or legs. Wriggling: forward belly slide movements. Pivoting: rotating sliding movements around navel axis.(Touwen 1976) | 5 – 9 months |
| Crawling | Abdominal crawling and/or crawling on four limbs.(Touwen 1976) | > 9 months |
| Sitting independently | Sitting without support > 1 minute.(Touwen 1976) | > 8 months |
| Standing independently | Standing up and stand free without support.(Touwen 1976) | > 12 months |
| Walking independently | Walking without support ≥ 7 paces consecutively.(Touwen 1976) | > 15 months |
| Toddling gait | Walking pattern with non-fluent, invariable movements, monotonous speed, block-like trunk movements, abducted shoulders and a broad gait width.(Hempel 1993) | 15 – 42 months |
| **Fine motor skills** |  |  |
| Pre-reaching phase | Arm extensions towards an object, with or without opening the hand.(Bremner and Wachs 2010) | 0 – 4 months |
| Reaching | Smooth approach of arms and hands towards an object, and touching or grasping the object.(Bremner and Wachs 2010) | > 4 months |
| Successful voluntary grasping | Grasping objects voluntary (not as a reflex).(Touwen 1976) | > 3 months |
| Palmar grasp | Grasp the object with whole palmar surface of hands and fingers.(Touwen 1976) | 0 – 4 months |
| Radial palmar grasp | Grasp the object with mainly the radial half of his palm, including thumb and index finger.(Touwen 1976) | 3 – 7 months |
| Scissoring grasp | Grasp the object between the volar surfaces of extended thumb and index finger.(Touwen 1976) | 7 – 10 months |
| Inferior pincer grasp | Grasp the object between the tip of index finger and volar side of the thumb.(Touwen 1976) | 8 – 12 months |
| Pincer grasp | Grasp the object neatly between the tips of index finger and thumb.(Touwen 1976) | > 11 months |

**Appendix S2. Description of assessed movement disorders**

| **Movement disorder** | **Description** |
| --- | --- |
| Ataxia | A movement disorders characterized by an impairment of the smooth performance of goal-directed movements, resulting in impaired ‘unconscious’ decision making about balance, speed, force and direction of intended movements.(Ghez and Thach 2000; Lawerman et al. 2015; Mumenthaler and H 2006) |
| Dystonia | A movement disorder characterized by sustained or intermittent muscle contractions causing abnormal, often repetitive, movements and/or postures.(Albanese et al. 2013) |
| Chorea | A movement disorders characterized by ongoing, random-appearing sequence of one or more discrete involuntary movements or movement fragments.(Sanger et al. 2010) |
| Myoclonus | A movement disorder characterized by a sequence of repeated, often non-rhythmic, brief shock-like jerks due to sudden involuntary contraction or relaxation of one or more muscles.(Sanger et al. 2010) |
| Tremor | A movement disorder characterized by a rhythmic back-and-forth or oscillating involuntary movement about a joint axis.(Sanger et al. 2010) |
| Tics | A movement disorder characterized by repeated, individually recognizable, intermittent movements or movement fragments that are almost always briefly suppressible and are usually associated with awareness of an urge to perform the movement.(Sanger et al. 2010) |
| Hypotonia | A decreased resistance to passive movement in rest, but with the ability to generate full force with active movements.(Sanger et al. 2010) |
| Movement disorder features | Physiological movement features of healthy children that resemble characteristics of movement disorders, according to above described definitions. These features are not phenotyped as *pathologic.* |
| Dystonic features | These physiologic movement features may resemble dystonia, such as the ATNR, inverse posturing of the feet, manipulation of objects, overflow movements and grimacing movements of the mouth. |
| Ataxic features | These physiologic movement features may resemble ataxia, such as suboptimal coordination during sitting, standing, walking and coordinated hand movements. |
| Choreatic features | These physiologic movement features may resemble chorea, such as the restless, smoothly rounded movements of fidgety GMs. |
| Myoclonic features | These physiologic movement features may resemble myoclonus, such as a quick, shock-like generalized movement (i.e. startle). |

**Appendix S3. Assessment form for the phenotypic appearance of movement disorder features**

Name observer:……………………………………………………

Date:………………………………………………………………

Patient number: …………………………………………………..

*Combined: in task specific, you may perceive different “main” features during different tasks, please describe:

A. task………………………………. Main feature…………………………….

B. task………………………………. Main feature…………………………….

If you perceive movement disorder features, please indicate for each:

1. task relatedness (if so, which); 2. body region; 3. global time indication
